# Loss of 15-Lipoxygenase in Retinodegenerative RCS Rats

**DOI:** 10.3390/ijms25042309

**Published:** 2024-02-15

**Authors:** Andrew James Mead, Kabir Ahluwalia, Brandon Ebright, Zeyu Zhang, Priyal Dave, Zeyang Li, Eugene Zhou, Aditya Anil Naik, Rachael Ngu, Catherine Chester, Angela Lu, Isaac Asante, Dimitrios Pollalis, Juan Carlos Martinez, Mark Humayun, Stan Louie

**Affiliations:** 1 Mann School of Pharmacy and Pharmaceutical Sciences, University of Southern California, Los Angeles, CA 90089, USA; amead@usc.edu (A.J.M.); kahluwal@usc.edu (K.A.); bebright@usc.edu (B.E.); zeyuz@usc.edu (Z.Z.); priyalda@usc.edu (P.D.); zeyangli@usc.edu (Z.L.); eugenezh@usc.edu (E.Z.); aanaik@usc.edu (A.A.N.); rngu@usc.edu (R.N.); chesterc@usc.edu (C.C.); alu52904@usc.edu (A.L.); asante@usc.edu (I.A.); 2 University of Southern California Ginsburg Institute for Biomedical Therapeutics, University of Southern California, Los Angeles, CA 90033, USA; pollalis@usc.edu (D.P.); juan.martinez@med.usc.edu (J.C.M.); humayun@med.usc.edu (M.H.); 3 University of Southern California Roski Eye Institute, Department of Ophthalmology, Keck School of Medicine, University of Southern California, Los Angeles, CA 90033, USA

**Keywords:** 15-lipoxygenase, specialized pro-resolving mediators, inflammation, retinitis pigmentosa, oxidative stress, Royal College of Surgeons rat, retinal degeneration

## Abstract

Retinitis pigmentosa (RP) is a retinal degenerative disease associated with a diversity of genetic mutations. In a natural progression study (NPS) evaluating the molecular changes in Royal College of Surgeons (RCS) rats using lipidomic profiling, RNA sequencing, and gene expression analyses, changes associated with retinal degeneration from p21 to p60 were evaluated, where reductions in retinal *ALOX15* expression corresponded with disease progression. This important enzyme catalyzes the formation of specialized pro-resolving mediators (SPMs) such as lipoxins (LXs), resolvins (RvDs), and docosapentaenoic acid resolvins (DPA RvDs), where reduced *ALOX15* corresponded with reduced SPMs. Retinal DPA RvD2 levels were found to correlate with retinal structural and functional decline. Retinal RNA sequencing comparing p21 with p60 showed an upregulation of microglial inflammatory pathways accompanied by impaired damage-associated molecular pattern (DAMP) clearance pathways. This analysis suggests that ALXR/FPR2 activation can ameliorate disease progression, which was supported by treatment with an LXA4 analog, NAP1051, which was able to promote the upregulation of *ALOX12* and *ALOX15*. This study showed that retinal inflammation from activated microglia and dysregulation of lipid metabolism were central to the pathogenesis of retinal degeneration in RP, where ALXR/FPR2 activation was able to preserve retinal structure and function.

## 1. Introduction

Retinitis pigmentosa (RP) is a heterogeneous retinal degenerative disorder linked to more than 40 genes [1,2]. Despite the differences in genetic origins, RP has a common molecular underpinning of chronic inflammation and oxidative stress. Cytokine profiling of ocular humors has revealed increases in inflammatory cytokines such as IL-1β and IL-6 in RP patient ocular humors [3]. In addition, ocular inflammation was accompanied by oxidative stress, which can increase blood–retinal barrier permeability [4].

To characterize the molecular pathways contributing to RP disease progression, a study evaluating the molecular changes between p21 and p60 in relation to retinal structural and functional changes was conducted. This study dissected ocular changes in RCS rats using a targeted LC-MS/MS lipidomics assay and gene expression analyses from p21 baseline to p60. To further interrogate the molecular mechanism(s) driving disease pathogenesis, this study probed whether activation of ALXR/FPR2 could mediate restoration of homeostasis of lipid metabolism and inflammation, as described previously in an alcoholic hepatitis animal model [5].

To investigate the effects of ALXR/FPR2 activation in the retinal microenvironment, a metabolically stable small molecule lipoxin A4 (LXA4) analog, NAP1051, was administered to two RP animal models, the RCS rat and the *rd10* mouse. The structure of NAP1051 has been previously published by Dong et al. [6]. The MerTK mutation of RCS rats impairs phagocytosis, where MerTK is required for the recognition and clearance of rod outer segment (OS) debris and apoptotic cells, resulting in the apoptosis and necrosis of rod photoreceptors (PRs) [7]. The *rd10* mouse features a mutation of the phosphodiesterase 6b (*PDE6B*) subunit, which leads to increased cGMP levels and Ca^2+^ accumulation, which can initiate apoptosis via ER stress pathways or calpain-mediated activation of apoptosis-inducing factor (AIF) and caspase-7 [8]. The *rd10* mouse model was selected as a second RP animal model, as opposed to the *rd1* RP mouse model with a more rapid rate of degeneration, due to a wider window for therapeutic intervention and a timeline of retinal degeneration that does not overlap with retinal development [9]. Using lipidomics, gene expression analysis, and RNA sequencing in the NPS, we dissected the molecular changes in the retinal microenvironment with advancing degeneration in RCS rats. Then, we evaluated the effects of intravitreal (IVT) administration of NAP1051 in the RCS rat and developed an oral formulation of NAP1051 for testing in both the RCS rat and *rd10* mouse.

## 2. Results

### 2.1. Ocular PUFAs and Their Metabolites in Relation to Retinodegeneration

**Figure 1:**
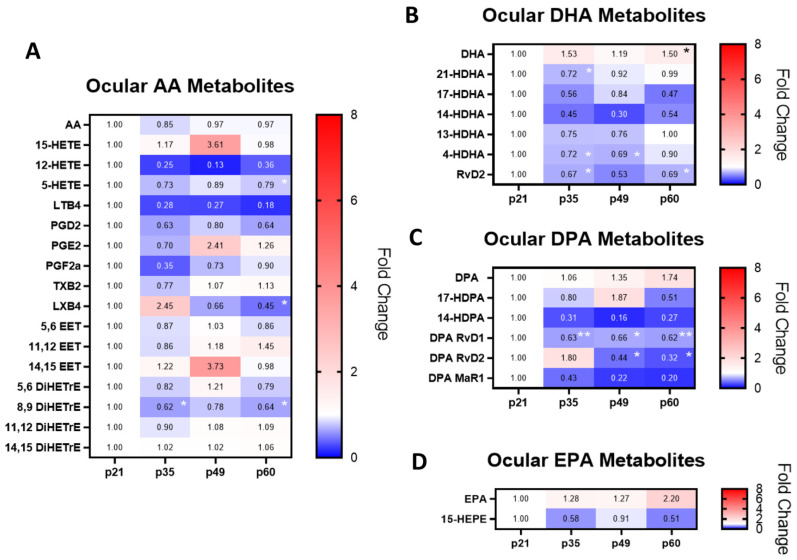
Targeted ocular lipidomics analyses of PUFAs and their metabolites over the disease course. The ω-6 PUFA arachidonic acid (AA) was relatively unchanged in relation to age (**A**). However, the AA metabolites 5-HETE, LXB4, and 8,9 DiHETrE were reduced on p60. In contrast, ω-3 PUFAs DHA, DPA, and EPA were increased with retinal degeneration (**B**–**D**). Despite ω-3 PUFA increases, their metabolites were reduced over the disease course, where reductions in SPMs such as DHA RvD2, DPA RvD1, and DPA RvD2 were identified. The fold change differences in ocular PUFAs and their metabolites were normalized to baseline p21 levels and compared to p35, p49, and p60 levels (*n* = 3 each group, * *p* < 0.05, ** *p* < 0.01, Kruskal–Wallis with Dunn’s correction).

In a previously reported RCS disease progression study, increased retinal inflammation and oxidative stress corresponded with retinal structural and functional declines [10]. This study showed an accumulation of retinal oxidative products such as malonaldehyde (MDA) and 4-hydroxy-2-nonenal (4HNE), which corresponded with reductions in PR counts and retinal thickness. Correlations between MDA and 4HNE also corresponded with increased PAD4 activity and increased retinal protein citrullination. To determine the effects of increased oxidative stress on polyunsaturated fatty acid (PUFA) levels, a targeted lipidomics assay quantifying PUFAs and their respective metabolites was conducted. Ocular tissues were collected on postnatal day 21 (p21), p35, p49, and p60 for bioanalysis, and the lipid levels were summarized in Figure 1. Changes in PUFAs and their metabolites were expressed as fold changes as compared to baseline ocular levels at p21.

Changes in the levels of the omega-6 (ω-6) PUFA arachidonic acid (AA) and its metabolites were evaluated across disease progression (Figure 1A). Retinal AA levels were unchanged throughout the disease course, where the range of expression was 0.85- to 0.97-fold of p21 levels. However, reductions in AA metabolites were observed on p60, where declines in 5-HETE, LXB4, and 8,9 DiHETrE were found (* *p* < 0.05, ** *p* < 0.01, Kruskal–Wallis with Dunn’s correction). Interestingly, levels of retinal LXB4, an SPM, were increased at p35 to 2.45-fold of p21 levels but dropped below p21 levels at p49 and p60. Other notable increases in AA metabolites included 15-HETE, PGE2, and 14,15 EETs, where these metabolites were 3.61-, 2.41-, and 3.73-fold higher than p21 levels at p49, respectively. However, these changes were not statistically significant.

A balance between pro-resolving and inflammatory lipids is key to maintaining inflammatory and pro-resolving homeostasis. In this context, ocular omega-3 (ω-3) fatty acids such as docosahexaenoic (DHA), docosapentaenoic acid (DPA), and eicosapentaenoic acid (EPA) were all increased during the retinal degenerative process, reaching maximal levels at p60. These findings showed that retinal degeneration corresponded to a 1.5- to 2.20-fold increase in the ω-3 PUFAs on p60 when compared to p21. Despite increases in ω-3 PUFA levels throughout the disease course, their respective metabolites were all predominantly reduced, suggesting metabolic dysregulation of ω-3 PUFAs and the formation of downstream SPMs (Figure 1B–D). At early stages of retinal degeneration at p35, 21-HDHA, 4-HDHA, DHA RvD2, and DPA RvD1 were all statistically significantly reduced (* *p* < 0.05, ** *p* < 0.01). Interestingly, there was an increase in DPA RvD2 levels at p35, which was similar to the trend observed for the ω-6 metabolite LXB4.

Ocular DHA was increased across the entire spectrum of the disease course, where this increase was statistically significant at p60 (* *p* < 0.05; Figure 1B). Despite an increase in DHA, the mono-hydroxylated metabolites such as 4-HDHA were all reduced by p35. 17-HDHA and its metabolite RvD2 were decreased at all time points, though only the decreases in RvD2 were statistically significant on p35 and p60 (* *p* < 0.05).

Similarly, DPA was also increased during the retinodegenerative process, and its mono-hydroxylated metabolites such as 14- and 17-HDPA were reduced at p35 (Figure 1C). Similarly, the SPM metabolite DPA RvD1 was also consistently reduced throughout the disease course (* *p* < 0.05, ** *p* < 0.01). While DPA RvD2 was increased on p35, its levels dropped below p21 levels at p49 and p60 (* *p* < 0.05).

### 2.2. Correlations of Ocular PUFA Metabolites with Changes in Retinal Structure and Function

**Figure 2:**
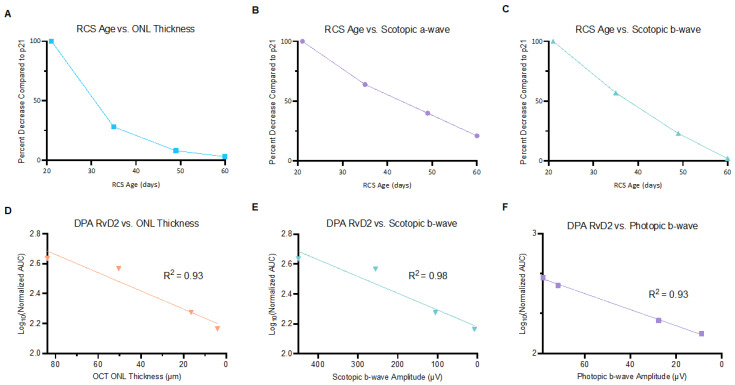
Correlations of ocular PUFA metabolites, retinal structure, and function. Retinal thickness as measured by OCT (**A**) and scotopic a- and b-waves plotted in relation to RCS rat age (**B**,**C**). DPA RvD2 demonstrated statistically significant Pearson’s correlations with declines in ONL thickness (R^2^ = 0.93), scotopic b-wave (R^2^ = 0.98), and photopic b-wave recordings (R^2^ = 0.93) (**D**–**F**) (*n* = 3 each time point).

To determine whether changes in the PUFAs and their metabolites correlated with structural and functional outcomes in RP animal models, a Pearson correlation matrix was generated relating changes in log-transformed PUFA metabolite peak areas with mean electroretinogram (ERG) recordings or mean ocular coherence tomography (OCT) measurements from p21 to p60 (Figure 2). ERG recordings involve stimulation of the retina in dark-adapted conditions (scotopic) and light-adapted conditions (photopic), where scotopic recordings are related to rod PR function and photopic recordings are related to cone PR function [11]. OCT is used to measure retinal structure changes in relation to time.

Aging of RCS rats correlated with reductions in ERG and OCT measurements. Outer Nuclear Layer (ONL) thickness (Figure 2A) as well as ERG scotopic a- and b-wave amplitudes were reduced in relation to increasing RCS rat age (Figure 2B,C). Ocular ERG/OCT recordings were correlated to log-transformed peak areas of ocular lipid metabolites. Declines in ONL thickness, scotopic b-wave, and photopic b-wave showed linear correlations with declines in ocular DPA RvD2 (Figure 2D–F). These findings suggest that DPA RvD2 may be a critical marker of retinal structural and functional status.

### 2.3. Ocular Metabolite Ratios and Retinal Lipoxygenase Gene Expression

**Figure 3:**
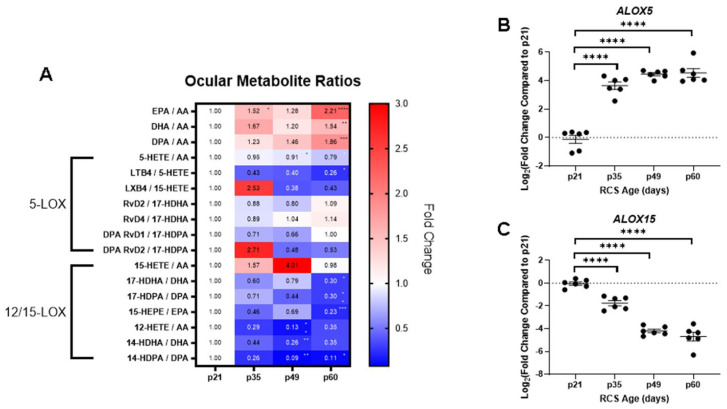
Retinal PUFA metabolite ratios and lipoxygenase gene expression. A heatmap displaying ocular PUFA metabolite ratios in relation to p21 (**A**). Ratios were analyzed via the Kruskal–Wallis test with Dunn’s correction (* *p* < 0.05, ** *p* < 0.01, *** *p* < 0.001, **** *p* < 0.0001). Retinal gene expression of *ALOX5* (**B**) and *ALOX15* (**C**) in relation to postnatal ages. *ALOX5* gene expression was upregulated in relation to RCS age, and *ALOX15* gene expression was downregulated in relation to RCS age. Data represented as median ± 95% CI, **** *p* < 0.0001, two-way ANOVA with Tukey’s correction.

To further dissect retinal lipidome dynamics, PUFA and metabolite ratios were generated from the lipidomics data and plotted in a heatmap (Figure 3A). The ratios between the ω-3 and ω-6 PUFAs, SPMs, and their precursors were calculated at each time point relative to baseline ratios. The ω-3/ω-6 PUFA ratios were consistently increased with disease progression, where the ratios of DHA, DPA, and EPA to AA were all statistically significantly higher at p60 relative to p21 (** *p* < 0.01 to **** *p* < 0.0001, Kruskal–Wallis test with Dunn’s correction). This finding suggests that there is an accumulation of the ω-3 PUFAs with disease progression since the AA levels were unchanged across the time points.

In addition, the ratios of PUFA metabolites formed through ALOX5-mediated metabolism were reduced for the majority of the time points. Exceptions included LXB4:15-HETE and DPA RvD2:17-HDPA ratios at p35. Despite an increase in LXB4:15-HETE at p35, this ratio decreased at p49 and p60. These ratios correlated with increases in *ALOX5* expression at p35, which metabolizes the precursors to these SPMs (Figure 3B).

Product over reactant ratios for 12/15-LOX metabolism were calculated, where most of these ratios were decreased throughout the disease course. Statistically significant declines in these ratios were found at p49 and p60, where the mono-hydroxylated metabolites of AA and the ω-3 PUFAs were reduced (* *p* < 0.05, ** *p* < 0.01, *** *p* < 0.001). The reduction in these metabolites corresponded with decreased *ALOX15* expression (Figure 3C). *ALOX15* expression declined between p21 and p49, when peak retinal degeneration occurred and plateaued between p49 and p60. The reductions in retinal *ALOX15* identified by gene expression analysis corresponded with the loss of pro-resolving SPMs and their upstream intermediates identified from lipidomics analyses.

### 2.4. RNA Sequencing and Ingenuity Pathway Analysis Predicts Declines of SPMs with Disease Progression in RCS Rats

Retinal tissue RNA sequencing was conducted at p21 (*n* = 3) and compared to p60 (*n* = 3) to identify gene expression changes in relation to disease progression. The RNA sequencing data were subsequently analyzed by ingenuity pathway analysis (IPA) to identify the regulatory pathways involved in the identified retinal gene expression changes. From these analyses, a loss of SPMs, increasing retinal inflammation with disease progression, and dysfunctional lipid metabolism were identified.

**Figure 4:**
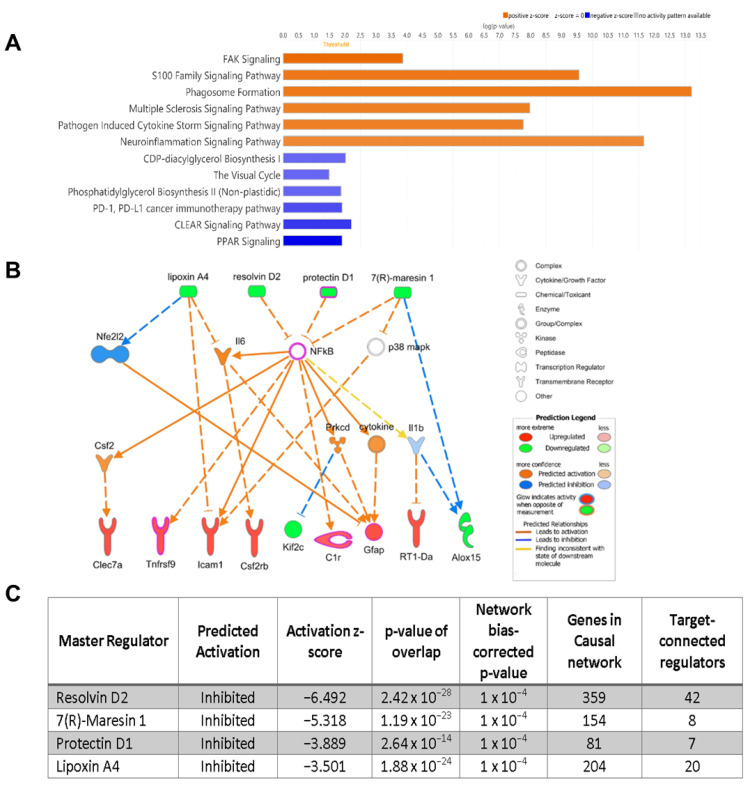
RNA sequencing and IPA analysis. RNA sequencing analysis comparing p21 with p60 shows upregulation (orange) of FAK, S100, phagosome formation, and neuroinflammation pathways as well as downregulation (blue) of visual cycle, PPAR, and CLEAR pathways (**A**). Positive and negative z-score values were used to identify the most relevant pathway changes in RCS rats. Ingenuity pathway analysis (IPA) of RNA sequencing predicted network of upstream mediators regulating the RNA sequencing outcomes (**B**). IPA of RNA sequencing identified resolvin D2 (RvD2), maresin 1 (MaR1), protectin D1 (PD1), and lipoxin A4 (LXA4) as master regulators (**C**).

Retinal cells were labeled by cell type and analyzed by RNA sequencing at p21 baseline and p60 [12] (Figure 4A). This analysis showed that visual cycle pathways were modulated in PRs, where the abundance and functionality of PRs declined with disease progression. The microglial dataset displayed upregulated neuroinflammation, phagosome formation, S100 proteins, focal adhesion kinase (FAK) signaling pathways, downregulation of peroxisome proliferator-activated receptors (PPARs), and coordinated lysosomal expression and regulation (CLEAR) signaling pathways.

S100 proteins are DAMPs that are released by damaged PRs, which contribute to sterile inflammation [13] and elicit pro-inflammatory cytokine release through binding onto RAGE and TLR-4 receptors, which activate NF-κB [14]. FAK, a tyrosine kinase, is associated with cell adhesion, motility, and proliferation, and its activation is associated with TLR-4 activation, which leads to NF-κB stimulation [15].

PPARs are transcription factors that play a critical role in lipid metabolism, such as oxidative degradation of fatty acids, as well as regulate inflammation through inhibition of NF-κB through ALXR/FPR2 stimulation [16,17]. In this analysis, the CLEAR signaling pathways were downregulated, where these genes are involved in lysosomal function, autophagy, and phagocytosis [18]. These findings from RNA sequencing indicated an increase in pro-inflammatory signaling accompanied by disrupted clearance pathways.

To further investigate the results from RNA sequencing, IPA of upstream signaling components was conducted, which identified the critical regulators of inflammatory outcomes (Figure 4B). Specific genes identified from this analysis showed upregulated lectins (*Clec 7a*), cytokine receptors (*tnfrsf9*, *csf2rb*), and *GFAP*. Clec 7a has been identified as a marker of neurodegenerative microglia in the retinas of Alzheimer’s mouse models, where the ablation of Clec 7a microglia preserved blood–retinal barrier function and reduced retinal inflammation [19]. Activation of microglia via stimulation of TNFRSF9 has been associated with neuroinflammation via the killing of oligodendrocytes [20], and CSF2 over-expressing microglia from retinal explants featured increased proliferation, migration, and phagocytosis [21]. GFAP is upregulated during the death of photoreceptors or mechanical injury and is indicative of retinal stress [22]. Furthermore, the analysis confirmed our findings that *ALOX15* gene expression was downregulated (Figure 3A), where microglial *ALOX15* was found to be reduced (Figure 4B). The IPA upstream analyses showed that the production of the PUFA metabolites lipoxin A4, resolvin D2, protectin D1, and 7(R)-maresin 1 were predicted to be inhibited (Figure 4C).

### 2.5. NAP1051 IVT Administration Demonstrates Preservation of ONL Nuclei and Reductions in PAD4

**Figure 5:**
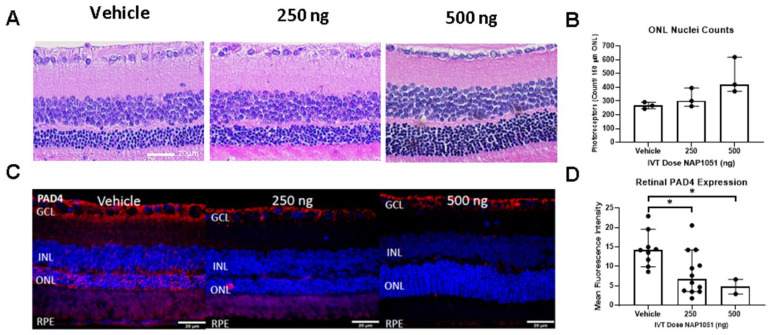
ONL PR counts and retinal PAD4 immunofluorescence staining. IVT NAP1051 administration promotes PR preservation in a dosage-dependent manner (**A**), which was correlated with PR counts (**B**). Adjacent histological sections were stained with anti-PAD4 (red) antibodies and DAPI (blue) (**C**), which showed reduced retinal PAD4 fluorescence intensity (**D**). Data represented as median ± 95% CI, * *p* < 0.05, Kruskal–Wallis with Dunn’s correction.

The IPA results suggested that LXA4 may play a role in the activation of microglial inflammation, whereas others have shown that ALXR/FPR2 signaling can interact with 12/15-LOX activity [23]. To evaluate the effects of NAP1051, a metabolically stable LXA4 analog, a proof-of-concept study was conducted with intravitreal (IVT) injection of NAP1051 to RCS rats starting at p21. Animals were stratified to receive vehicle, 250 ng, or 500 ng IVT NAP1051 doses on p21 and p35, with the end of the study on p49. Histological assessment at p49 of RCS rat retinas indicated that at the 500 ng dosage, ONL PRs were preserved, though not to a statistically significant level (Figure 5A,B). To determine if IVT NAP1051 was correlated with reductions in molecular markers of inflammation, immunofluorescence (IF) staining of PAD4 in RCS retinas was conducted (Figure 5C) and the percent area of retinal PAD4 fluorescence was quantified (Figure 5D). PAD4 is an enzyme involved with protein citrullination and retinal gliosis, where it has been previously shown that increases in PAD4 and protein citrullination occur in relation to retinal degeneration [10].

Red PAD4 staining was detected in the GCL, ONL, and OS layers of the vehicle-treated RCS rats. However, retinas treated with 250 ng NAP1051 showed reductions in PAD4 staining of the ONL and OS layers. PAD4 staining was undetectable outside of the GCL in animals treated with 500 ng NAP1051.

### 2.6. Oral NAP1051 Administration Preserved Retinal Structure and Function

**Figure 6:**
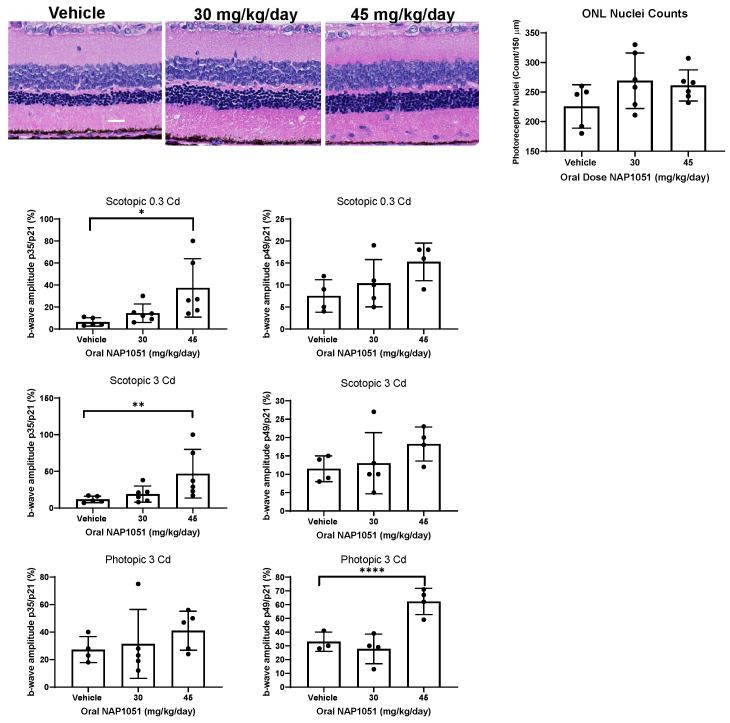
Oral administration of NAP1051 preserved retinal structure and function. RCS rats treated with NAP1051 given orally daily from p21 to p49 showed PR preservation (**A**), where the counts of PR nuclei from 150 µm segments of the ONL were enumerated (**B**). ERG was conducted on p21, p35, and p49, and the b-wave amplitudes were plotted in relation to the p21 baseline b-wave amplitudes (**C**,**D**). Statistically significant preservation of the scotopic ERG response at 0.3 and 3 Cd for the 45 mg/kg/day dosage group at p35 was identified. At the p49 follow-up, photopic ERG preservation at the 3 Cd was demonstrated for the group receiving 45 mg/kg/day. Data represented as mean ± SD, * *p* < 0.05, ** *p* < 0.01, **** *p* < 0.0001, 2-way ANOVA test with Dunnett’s correction.

While the IVT NAP1051 study demonstrated that the therapeutic intervention could exert its pro-resolving activities when administered IVT, this administration limits the number of dosages. Therefore, NAP1051 was administered orally at 30 mg/kg/day and 45 mg/kg/day and compared to vehicle (Figure 6A). Oral NAP1051 was able to preserve the ONL in comparison to the vehicle, although the degree of preservation was not statistically significant (Figure 6B).

Despite the lack of statistically significant PR preservation, oral NAP1051 was able to preserve ocular function in a dosage-dependent manner as measured by ERG. The scotopic 0.3 Cd, scotopic 3 Cd, and photopic 3 Cd flash intensities show rod PR function, mixed rod and cone function, and cone function, respectively (Figure 6C,D). Statistically significant preservation of the scotopic 0.3 Cd and scotopic 3 Cd b-wave amplitudes was identified at p35 in relation to the vehicle, (* *p* < 0.05; ** *p* < 0.001, Kruskal–Wallis test with Dunn’s correction; Figure 6C), in addition to significant preservation of the photopic 3 Cd b-wave amplitudes on p49 in RCS rats given 45 mg/kg/day (**** *p* < 0.0001; Figure 6D). These findings may represent the early decline of rod PRs seen in RP, where cone PRs are subsequently impacted by the death of surrounding rod PRs [24]. NAP1051 was able to preserve rods in the early stages of retinal degeneration as shown in the scotopic findings at p35 (Figure 6C), as well as delay cone declines as evidenced by the preservation of the photopic 3 Cd ERG at p49 (Figure 6D).

### 2.7. Effects of NAP1051 on Ocular Gene Expression

**Figure 7:**
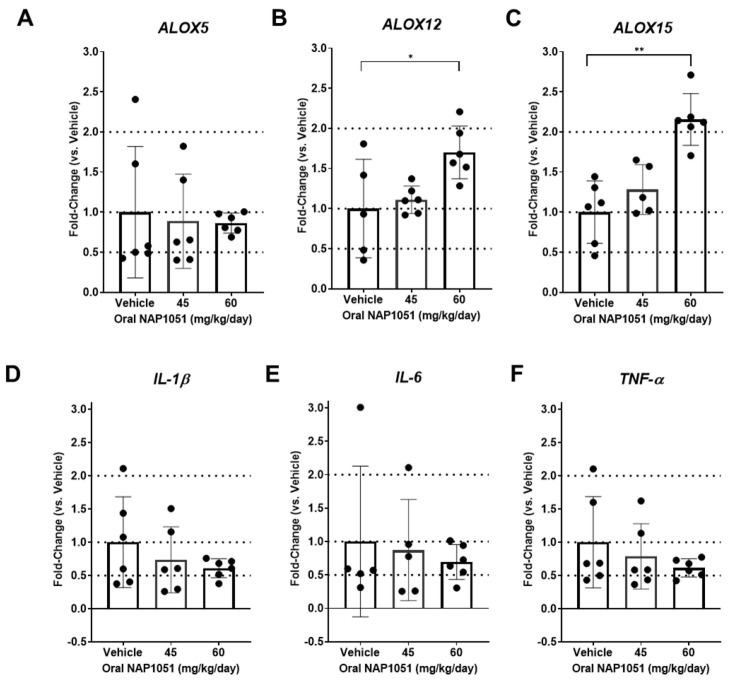
Impact of NAP1051 on retinal gene expression on p49. There was a dosage-dependent increase in *ALOX12* and *ALOX15* expression (**B**,**C**), but no changes in *ALOX5* (**A**), where statistically significant increases were observed for 60 mg/kg/day. Reductions in *IL-1β*, *IL-6*, and *TNF-α* gene expression were identified, but these changes were not statistically significant (**D**–**F**). Data represented as mean ± SD, * *p* < 0.05, ** *p* < 0.01, Kruskal–Wallis test with Dunn’s correction * *p* < 0.05, ** *p* < 0.01. Dotted lines were included at fold-change values of 0.5, 1, and 2 for ease of comparison between gene expression levels.

Encouraging results in retinal structural and functional preservation (Figure 6) after oral administration of NAP1051 prompted a molecular investigation into the mechanism of action. Retinal function improvements in RCS rats treated with 45 mg/kg/day prompted dosage escalation to 60 mg/kg/day to determine if a higher dosage would further improve retinal preservation. Retinal gene expression of RCS rats treated with vehicle, 45 mg/kg/day, and 60 mg/kg/day of NAP1051 at p49 showed a dosage-dependent increase in *ALOX12* and *ALOX15*, but not *ALOX5* (* *p* < 0.05, ** *p* < 0.01, Kruskal–Wallis test with Dunn’s correction; Figure 7A–C). NAP1051 treatment reduced inflammatory cytokine gene expression of *IL-1β*, *IL-6*, and *TNF-α*, however, these changes were not statistically significant (Figure 7D–F).

### 2.8. NAP1051 Impact on Lipidomics Analyses of RCS Rat Ocular Lipids

**Figure 8:**
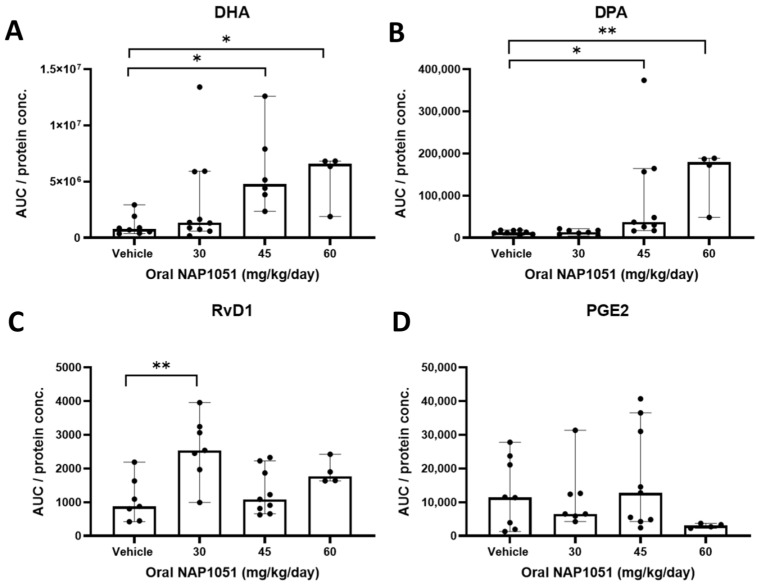
Effect of NAP1051 on retinal lipidomics. A dosage-dependent increase in ω-3 PUFAs such as DHA (**A**) and DPA (**B**) was identified at 45 mg/kg/day and 60mg/kg/day as compared to vehicle treatment. Metabolites of ω-3 showed an increase in ocular RvD1 levels for animals treated with 30 mg/kg/day, but not at 45 mg/kg/day or 60 mg/kg/day (**C**). These findings were accompanied by reduction in ocular levels of PGE2 at 60 mg/kg/day, but not to statistically significant levels (**D**). Data represented as median ± 95% CI, * *p* < 0.05, ** *p* < 0.01, Kruskal–Wallis test with Dunn’s correction.

To assess changes in the ocular lipidome in relation to oral NAP1051 administration, ocular biolipid products from RCS rats treated with oral NAP1051 were evaluated via the LC-MS/MS lipidomics assay (Figure 8). NAP1051 was able to significantly increase the ω-3 PUFAs DHA and DPA at the 45 mg/kg/day and 60 mg/kg/day dosages (* *p* < 0.05, ** *p* < 0.01, Kruskal–Wallis test with Dunn’s correction; Figure 8A,B). Interestingly, 30 mg/kg/day NAP1051 was able to increase RvD1 levels in the retina (** *p* < 0.01, Figure 8C). Furthermore, NAP1051 was able to reduce PGE2, a pro-inflammatory ω-6 metabolite, at 60 mg/kg/day. However, this reduction was not statistically significant.

### 2.9. Oral NAP1051 Administration to rd10 Mice Preserved Retinal ONL PRs, Reduced FAF Appearance, and Reduced Markers of Inflammation and Oxidative Stress

**Figure 9:**
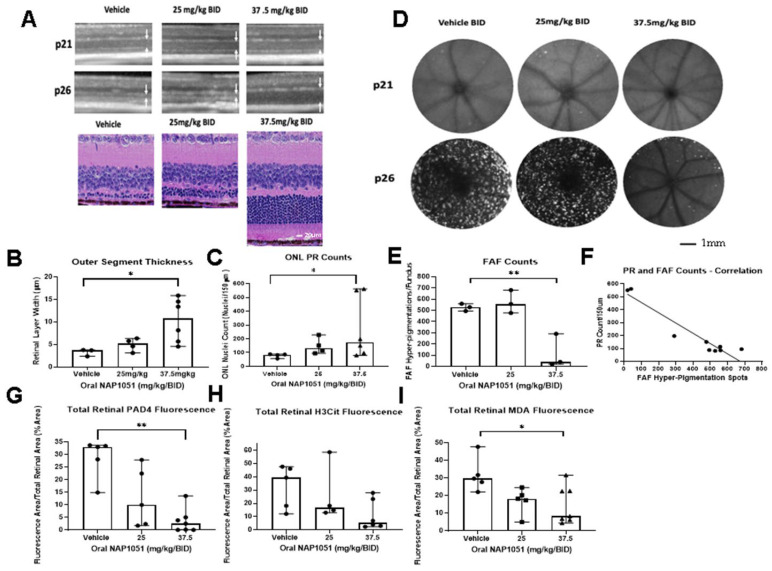
Effects on *rd10* mice treated with NAP1051. *rd10* mice treated with NAP1051 from p15 to p26 showed ONL preservation on OCT (arrows) and H&E on p26 (**A**). NAP1051 was able to preserve ONL thickness (**B**) and PR counts (**C**). OCT-FAF showed NAP1051 reduced hyperpigmentation on p26 (**D**,**E**). Correlations between the ONL PR counts and FAF hyperpigmentation were evaluated and demonstrated a negative linear correlation (R^2^ = 0.9) where fewer hyperpigmentation spots were correlated to increased ONL PR nuclei (**F**). Retinal PAD4 immunofluorescence staining was reduced for animals treated with 37.5 mg/kg/BID (**G**) corresponding with reduced retinal H3Cit, but not to a statistically significant level (**H**). The oxidative stress marker malondialdehyde (MDA) was statistically significantly reduced at the 37.5 mg/kg/BID dosage (**I**). Data represented as median ± 95% CI, * *p* < 0.05, ** *p* < 0.01, Kruskal–Wallis test with Dunn’s correction. The number of PRs in the ONL and hyperpigmentation spots seen in FAF were enumerated using ImageJ/FIJI (ver 2.3.0).

Structural, molecular, and functional findings from IVT and oral administration of NAP1051 to RCS rats were encouraging. However, it is not certain whether its effects are limited to the MerTK mutation of RP found in RCS rats. To analyze the effects of NAP1051 in a second animal model, NAP1051 was administered orally to *rd10* mice. The *rd10* mouse has a *PDE6B* mutation, which results in excessive Ca^2+^ accumulation, promoting subsequent rod apoptosis [25]. *rd10* mice were administered vehicle, 25 mg/kg, and 37.5 mg/kg of NAP1051 by oral gavage twice daily (BID) beginning on p15 until the end of the study on p26. Dosages were determined based on guidance for allometric scaling between rats and mice [26]. Retinal structures were evaluated with OCT, fundus auto-fluorescence (FAF), and histological assessment at p21 and p26 (Figure 9). Oral NAP1051 administration at 37.5 mg/kg/BID resulted in the preservation of retinal structure (Figure 9A), where the thickness of the OS layer and PR counts in the ONL were statistically significantly preserved as compared to vehicle (* *p* < 0.05, Kruskal–Wallis test with Dunn’s correction; Figure 9B,C). These findings suggest that ONL thickness and the PR counts were preserved by oral NAP1051.

FAF hyperpigmentation can be visualized after blue light stimulation due to the accumulation of retinal fluorophores in phagocytic cells, which are indicative of phagocytic dysfunction from damaging oxidative stress [27]. FAF recordings at p21 and p26 (Figure 9D) revealed the development of FAF hyperpigmentation in both the vehicle and 25 mg/kg/BID NAP1051 groups on p26. In contrast, there was reduced FAF hyperpigmentation for the 37.5 mg/kg/BID treatment group at p26 (** *p* < 0.01; Figure 9D,E). Histological enumeration of PR counts at p26 indicated a strong negative correlation (R^2^ > 0.9) with FAF hyperpigmentation (Figure 9F). This finding indicates that FAF hyperpigmentation correlated with PR decline, where the 37.5 mg/kg/BID dosage group had significantly reduced FAF hyperpigmentation as well as preserved ONL PR counts.

NAP1051 reduced PAD4 and H3Cit IF staining, where PAD4 was statistically significantly reduced at the 37.5 mg/kg/BID dosage (** *p* < 0.01, Figure 9G). However, the downregulation of H3Cit did not achieve statistical significance (Figure 9H). The level of retinal oxidized lipids was evaluated using IF staining of malondialdehyde (MDA) to determine the effect of NAP1051 on retinal oxidative stress, where the 37.5 mg/kg/BID dosage was able to reduce retinal MDA staining (* *p* < 0.05; Figure 9I). These findings indicate that ALXR/FPR2 activation using NAP1051 was able to reduce retinal MDA and inhibit gliosis via the reduction in PAD4 activity and protein citrullination.

## 3. Discussion

In this study, retinal lipidomics, gene expression, and RNA sequencing were evaluated in RCS rats across the disease course. We then investigated the effects of ALXR/FPR2 stimulation on changes in retinal structure and function, ocular lipid dynamics, and markers of ocular oxidative stress and inflammation in the RCS rat and *rd10* mouse models of RP using the LXA4 analog NAP1051.

To understand the molecular mechanisms driving increased ω-3 PUFA ocular levels but declines in their downstream metabolites, product over reactant ratios were produced to identify potential metabolic dysfunction. The product over reactant ratios supported the finding that in early disease stages, elevated levels of ocular LXB4 and DPA RvD2 may be a consequence of increased ALOX5 expression to promote their metabolism. While the formation of DPA RvD2 is regulated upstream by the conversion of DPA to 17-HDPA by ALOX15, ALOX5 is required for the conversion of 17-HDPA to DPA RvD2 [28]. Retinal gene expression showed a four-fold increase in *ALOX5* expression at p35 as compared to p21, which supported the finding of increased levels of LXB4 and DPA RvD2. However, at p49, reductions in *ALOX15* prevent the formation of precursors, which are metabolized to form DPA RvD2. Furthermore, gene expression analysis showed that there were reductions in *ALOX15* expression, which was significantly reduced by p60.

To further understand the molecular dynamics with disease progression, RCS retinal RNA sequencing results were compared between p21 and p60. It was found that microglial cell dysfunction may be central to the pathogenesis of RP. Neuroinflammation, phagosome formation, S100 proteins, and focal adhesion kinase (FAK) signaling pathways were upregulated, and PPAR and CLEAR signaling pathways were downregulated. These findings further support that ocular DAMPs and the inability to remove these factors contribute to sterile inflammation. Our findings also suggested that activation of ALXR/FPR2 could mitigate DAMP-mediated inflammation and oxidative stress. LXA4 signaling was predicted to be inhibited from the IPA prediction network, where LXA4 administration to RCS rats induced ocular RvD1 levels. RvD1 is a ligand for ALXR/FPR2, and its effects have been shown in an endotoxin-induced uveitis rat model where direct IVT administration of RvD1 improved clinical scores and reduced TNF-α and NF-κB activation [29]. The authors suggested that RvD1 was able to reduce M1 macrophages and increase M2 macrophage polarization. The role of ALXR/FPR2 activation was also shown in a cerebral ischemia–reperfusion injury model, where LXA4 induced microglial polarization towards an M2 phenotype that can more effectively remove DAMPs by efferocytosis [30]. Similarly, Queck et al. showed that ALOX12/15 knockout can exacerbate alcoholic hepatitis, while activation of ALXR/FPR2 using LXA4 can attenuate alcohol-induced hepatic steatosis in ALOX12/15^−/−^ mice [23]. Intraperitoneal administration of LXA4 in both ALOX12/15^−/−^ and ALOX12/15^+/+^ mice reduced hepatic immune cell infiltration and systemic inflammatory cytokines, which suggests that LXA4 administration was able to induce pro-resolving effects. ALOX15-deficient mice had impaired epithelial wound repair, and Gronert et al. demonstrated that treatment with LXA4 or NPD1 accelerated corneal re-epithelialization [31]. Furthermore, Calandria et al. demonstrated that silencing of ALOX15 in ARPE-19 cells increased oxidative stress-induced apoptosis of RPE cells, highlighting the importance of ALOX15 activity in cell survival in an oxidative stress milieu [32]. However, Calandria et al. showed that only NPD1, but not LXA4, was able to rescue 15-lipoxygenase-1 silenced RPE cells from oxidative stress-induced apoptosis. These results demonstrate that 15-lipoxygenase-1 is activated by oxidative stress in ARPE-19 cells and that NPD1 is part of an early survival signal in RPE cells.

Since retinal *ALOX15* expression was reduced, this study used NAP1051, a metabolically stable LXA4 analog, to determine its impact on disease progression in RCS rats. When RCS rats were administered NAP1051, structural and functional preservation of the retina was accompanied by increased ocular expression of *ALOX12* and *ALOX15*. Although the increase in *ALOX15* expression for the 45 mg/kg/day dosage group was not statistically significant (Figure 7C), the mean 1.28-fold increase in *ALOX15* expression was sufficient for the preservation of visual function as measured by ERG (Figure 6). This may be attributable to the direct anti-inflammatory and pro-resolving effects of NAP1051 outside of restoration of homeostasis of lipid metabolism, such as promotion of macrophage and microglial polarization and inhibition of pro-inflammatory cytokine release [33]. NAP1051 treatment was able to further increase ocular DHA and DPA levels. Interestingly, retinal RvD1 was only increased at 30 mg/kg/day of orally administered NAP1051 in RCS rats. This may be due to the ability of both RvD1 and NAP1051 to compete for ALXR/FPR2 binding [34], where higher NAP1051 dosages may downregulate ALXR/FPR2. ALXR/FPR2 activation using NAP1051 in *rd10* mice supported retinal PR preservation and reduced FAF hyperpigmentation, a sign of damage from oxidative stress. Reductions in oxidative stress were corroborated by reduced retinal MDA staining. Reductions in retinal PAD4 and H3Cit in this study are corroborated by previous findings, where increased retinal PAD4 and H3Cit expression correlate with retinal degeneration [10]. Taken together, this study demonstrated that dysregulated lipid metabolism is a consequence of ALOX15 depletion, which promotes damaging chronic inflammation from microglial activation. However, ALXR/FPR2 activation via NAP1051 was able to promote *ALOX12* and *ALOX15* expression and correlated with the preservation of retinal structure and function in RP models. These findings point to the critical role of ALOX15 in maintaining retinal structure and function.

This study featured certain limitations, including a lack of mouse ERG recordings, a narrower profile of identified lipids from the lipidomics assay in mice as opposed to rats, and limited studies of the nuances between the well-studied DHA metabolites and the less-studied DPA metabolites. Due to challenges in reliably registering *rd10* mice ERG recordings, the histological changes induced by oral NAP1051 could not be linked to the clinical outcome of the preservation of vision in mice, akin to the pairing of histological results and ERG recordings conducted in this study in the RCS rats. Furthermore, the small volume of the mouse vitreous fluid may have prevented comprehensive profiling of the mouse ocular lipidome, where fewer analytes were detected and at a lower intensity for the mice than the rats. Lastly, although DPA is a minor PUFA in relation to DHA in the retina, the loss of analytes such as DPA RvD1 and DPA RvD2 were found to be important factors in the progression of retinal degeneration. Although previous studies have investigated and discussed the roles of resolvins in the resolution of chronic inflammation [35,36], further studies are required to explore the nuances between the DHA and DPA metabolites and clarify their specific roles in the prevention of retinal degeneration.

## 4. Materials and Methods

### 4.1. Animals

Animal experiments were conducted according to protocols approved by the University of Southern California (USC) Institutional Animal Care and Use Committee (IACUC), the National Institute of Health Guide for the Care and Use of Laboratory Animals, and the Association for Research in Vision and Ophthalmology Statement for the Use of Animals in Ophthalmic and Vision Research. RCS rats were obtained from Dr. Matthew LaVail (University of California, San Francisco, CA, USA) and were bred at a USC vivarium according to approved IACUC protocols. The rats were housed in pathogen-free housing with water and food ad libitum on a 12 h light and dark cycle. Rat experiments were conducted between p21 and p60. The *rd10* mice were acquired from Charles River and similarly bred at the USC vivarium according to approved IACUC protocols. Oral gavage treatment of *rd10* was conducted from p15 to p26, and experiments were conducted on p21 and p26.

### 4.2. Natural Progression Study Design

The RCS rats for the natural progression study, without therapeutic intervention, had their ocular tissue collected and evaluated at p21, p35, p49, and p60. At p21 and end of study at p60, RNA sequencing, lipidomics, and gene expression were conducted. PUFA metabolite and gene expression evaluations were performed at p21, p35, p49, and p60. RNA sequencing was performed at p21 baseline and p60 end of study to assess the major gene expression changes at the beginning of disease manifestation and during advanced disease progression.

### 4.3. Retinal RNA Sequencing and IPA Analyses

Collected total RNA was evaluated for purity with a Nanodrop 2000 (Thermo Fisher, Waltham, MA, USA), Qubit (Invitrogen, Waltham, MA, USA), and TapeStation (Agilent, Santa Clara, CA, USA) systems to verify suitability for transcriptomics. Evaluated total RNA was sent to Azenta Life Sciences (South Plainfield, NJ, USA) for RNA sequence analysis using a HiSeq Illumina system (San Diego, CA, USA), and the data quality was evaluated using FastQC (ver 0.11.9). The analyzed read data were mapped to a reference genome (https://useast.ensembl.org/Rattus_norvegicus/Info/Index, accessed on 8 December 2022) for predictions of regulators of gene expression changes, using STAR [37]. FeatureCounts (ver 2.0.3) was used to produce count data from the RNA sequence reads, and DESeq2 (ver 1.26.0) was applied to normalize between the different time points. QIAGEN IPA (https://digitalinsights.qiagen.com/products-overview/discovery-insights-portfolio/analysis-and-visualization/qiagen-ipa/ accessed on 8 December 2022) was used to analyze the expressed genes between time points based on guidance pertaining to analysis approaches outlined in Kramer et al. [38].

### 4.4. Intravitreal NAP1051 Study Design

Intravitreal (IVT) injection of NAP1051 in RCS rats included the following treatment groups: (1) vehicle hydroxypropyl-β-cyclodextrin/HPBCD (Roquette Pharma, Clinton, IA, USA), (2) 250 ng, and (3) 500 ng NAP1051 formulated with HPBCD. Dosages of vehicle or NAP1051 were administered on p21 and p35, with end of study on p49. ERG recordings were conducted at p21, p35, and p49. NAP1051 was synthesized by Dr. Nicos Petasis at USC.

### 4.5. Electroretinogram (ERG) Recordings

ERG waveforms were recorded at baseline p21, p35, and p49. The changes over time were evaluated as the percentage of ERG waveform amplitudes preserved on p35 and p49 relative to baseline p21. RCS rats were first dark-adapted overnight prior to ERG recordings. Dark-adapted RCS rats were anesthetized via intraperitoneal injection with 80 mg/kg ketamine (Covetrus, Portland, ME, USA, Cat. # 071069) and 5 mg/kg xylazine (Vetone, Paris, France, Cat. # 510004) mixture, and ERG electrodes were fitted onto rat corneas. Conductivity between the cornea and the electrodes was maintained with ophthalmic gel (GenTeal Gel, NOVARTIS, Basel, Switzerland, containing hydroxypropyl methylcellulose). Dark-adapted scotopic recordings were conducted with an escalating range of flash intensities from 10 mCd to 25 Cd. After 10 min of light adaptation, photopic ERG recordings were conducted from an escalating range of light flash intensities from 100 mCd to 25 Cd. The recordings were analyzed in ERGVIEW (ver 4.380R, Ocuscience LLC, Kansas City, MO, USA) software. The 300 mCd and 3 Cd Scotopic ERG waveforms were filtered to include a-waves with implicit times from 25 to 50 ms, and b- waves with implicit times from 70 to 100 ms. For photopic ERG analysis, b-waves with implicit times between 50 and 75 ms were extracted. The Scotopic 3 Cd a-wave and b-wave amplitudes were plotted in Prism 8 (GraphPad, San Diego, CA, USA) for comparison.

### 4.6. Tissue Isolation and Preparation of Histological Sections

RCS rats and *rd10* mice were euthanized via isoflurane (Sigma-Aldrich, St. Louis, MO, USA, Cat. # 792632) inhalation in an isoflurane chamber followed by EuthaSol (Sigma-Aldrich, St. Louis, MO, USA, Cat. # P3761) infusion. Eyes designated for histological and immunofluorescence staining were placed in Davidson’s solution fixative (Fisher Scientific, Waltham, MA, USA, Cat. # 5029227) for 12 h, prior to transfer to 70% ethanol (VWR, Radnor, PA, USA, Cat. # 97064-490) and submission for histological sectioning. Sections were cut every 5–7 microns throughout the block. Then, 4–5 sections were mounted per slide. All slides were then stained with H&E. Images were taken on the Aperio CS2 (Leica Biosystems, Deer Park, IL, USA).

### 4.7. ImageJ/FIJI Quantification of H&E ONL Nuclei, FAF Hyperpigmentation, and Retinal Layer Thickness

H&E sections of the retina were imaged using a brightfield microscope at 40× magnification. To quantify the number of PRs and FAF hyperpigmentations, and measure retinal thicknesses, the image files were uploaded to ImageJ/FIJI (version 2.3.0) for analyses [39].

### 4.8. Immunofluorescent Staining

Retinal sections that were formalin-fixed in Davidson’s solution and paraffin-embedded onto tissue slides were stained for PAD4, H3Cit, and MDA according to a previous publication [40]. PAD4 rabbit anti-mouse primary antibody (Proteintech, Sankt Leon-Rot, Germany, Cat. # 17373-1-AP), H3Cit polyclonal rabbit anti-mouse (Abcam, Cambridge, UK, Cat. # ab5103), and MDA polyclonal rabbit anti-mouse (Abcam, Cambridge, UK, Cat. # ab27642) primary antibodies were diluted in blocking buffer and added to each retina and incubated overnight at 4 °C. The primary antibody dilutions were 1:100 for PAD4, 1:1000 for H3Cit, and 1:1000 for MDA. Secondary antibody of goat anti-rabbit IgG Alexa Fluor 594 (Thermo Fisher Scientific, Waltham, MA, USA, Cat. # A11037) was diluted in blocking buffer 1:500, and 30 μL was added to each retina, followed by a 45 min incubation at room temperature. An amount of 40 μL of Vectashield Vibrance Antifade Mounting Medium, with DAPI (Vector Laboratories, Newark, CA, USA, Cat. # H-1800-10) was used for mounting. Slides were visualized by immunofluorescent excitation at 595 nm.

### 4.9. RNA Extraction from Ocular Tissue

Ocular tissue was homogenized on the Tissuelyzer II (Qiagen, Hilden, Germany, Cat. # 85300) at 30 Hz for 5 min, followed by flipping of samples and an additional 5 min of lysis at 30 Hz. RNA was extracted from ocular tissue with Trizol reagent according to previously published methods [41]. RNA concentrations were read on a Nanodrop (Thermo Fisher Scientific, Wilmington, DE, USA).

### 4.10. cDNA Synthesis

A 1 μg amount of cDNA was generated from 1 μg of RNA in a 1:1 ratio according to manufacturer’s instructions with a Revertaid cDNA Synthesis Kit (Thermo Scientific, Waltham, MA, USA, Cat. # K1622) to a concentration of 1 μg cDNA/50 μL nuclease-free water. Tubes with cDNA synthesis mixtures were added into a T100 Thermal Cycler (Bio-rad, Hercules, CA, USA, Cat. # 1861096) and run according to the following program: (1) incubate 5 min at 25 °C, (2) incubate 60 min at 42 °C, (3) heat to 70 °C for 5 min to terminate reaction, and (4) hold at 4 °C indefinitely. Samples were diluted 1:10 with nuclease-free water to reduce the cDNA reaction components present in downstream qPCR applications. Diluted cDNA was plated into a 96-well plate to prepare for plating on a 384-well plate (VWR, Radnor, PA, USA) for qPCR.

### 4.11. Ocular qPCR

qPCR master mix was produced for the rat genes of *ALOX5*, *ALOX12*, *ALOX15*, *IL-1β*, *IL-6*, *TNF-α*, *β-Actin*, and *GAPDH* by mixing forward and reverse primers (Supplementary Table S1) with Power Up SYBR Green Master Mix (Applied Biosystems, Foster City, CA, USA). An 8 μL amount of master mix was pipetted in each well for each respective gene, followed by 2 μL of cDNA to plate 10 ng of cDNA per well (5 ng/μL cDNA). qPCR plates were centrifuged for 10 min at 2000× *g* to remove bubbles and bring qPCR reaction components into plate wells. The plate was run on the QuantStudio Flex 12K Flex Real-Time PCR System (Thermo Fisher Scientific, Waltham, MA, USA, Cat. # 4471087), and the run method was as follows: 1 cycle of 50 °C for 2 min, 1 cycle of 95 °C for 10 min, 40 cycles of 95 °C for 15 s for the melting step, and 60 °C for 1 min for the annealing/elongation step. Data were collected and analyzed in Data Assist (ver 3.01, Invitrogen, Carlsbad, CA, USA), where gene expression of target genes was normalized to GAPDH and β-Actin reference genes. Fold changes were plotted in GraphPad Prism 8 (GraphPad, San Diego, CA, USA).

### 4.12. LC-MS/MS Lipidomics Assay

The targeted PUFA lipidomics assay has been previously described by Ebright et al. [42].

### 4.13. Statistical Analyses

Both analytical and imaging data results were expressed as mean ± SD or median ± 95% CI unless specified otherwise. Shapiro–Wilk test was used for determination of normality. Statistical analyses used GraphPad Prism 8 (GraphPad, San Diego, CA, USA) for data preparation, statistical analyses, and data visualization on quantitative and qualitative data. Appropriate statistical analyses were performed for each data set. ANOVA or Kruskal–Wallis tests with multiple comparison corrections were used to determine the statistical significance, where the alpha was determined a priori to be set at a threshold of 0.05.

### 4.14. Ethical Approval

All animal experiments were approved by the Institutional Animal Care and Use Committee (IACUC) at the University of Southern California (USC). The IACUC has policies in place that are adherent to the National Institutes of Health (NIH) Guidelines for the Care and Use of Laboratory Animals. RCS rat and *rd10* mice colonies were bred and propagated at USC under IACUC-approved protocols.

## Data Availability

The transcriptomics data discussed in this publication have been deposited in NCBI’s Gene Expression Omnibus and are accessible through GEO Series accession number GSE237804 (https://www.ncbi.nlm.nih.gov/geo/query/acc.cgi?acc=GSE237804, accessed on 8 December 2022). The datasets used and/or analyzed during the current study are available from the corresponding author upon reasonable request.

## Supplementary Materials

The following supporting information can be downloaded at: https://www.mdpi.com/article/10.3390/ijms25042309/s1.
