# Supplementary material for: Loss of 15-Lipoxygenase in Retinodegenerative RCS Rats
Source: Int J Mol Sci. 2024 Feb 15;25(4):2309. doi: 10.3390/ijms25042309 (PMC10889776; doi:10.3390/ijms25042309)
Supplement: Supplementary file 1 [file ijms-25-02309-s001.zip › Supplementary Table.pdf]

qPCR Primers	Forward (5′ – 3′)<br>Reverse (3′ – 5′)
Rat ALOX5	ATGCCTTCCTACACTGTCACC<br>GAGCCAATGAGGCTGAGGTAA
Rat ALOX12	TCTCGGGATCCGTCAACCT<br>AATCAAACTCCTCCTCCTTGCC
Rat ALOX15	CTGTGGTTGGTTGGACAGCA<br>TTGAATTCTGCTTCCGAGTCCC
Rat IL-1β	CACCTCTCAAGCAGAGCACAG<br>GGGTTCCATGGTGAAGTCAAC
Rat IL-6	TCAACTCCATCTGCCCTTCAG<br>AAGGCAACTGGCTGGAAGTCT
Rat TNF-α	CGTCAGCCGATTTGCCATTT<br>TCCCTCAGGGGTGTCCTTAG
Rat β-Actin	GCAGGAGTACGATGAGTCCG<br>ACGCAGCTCAGTAACAGTCC
Rat GAPDH	AGTGCCAGCCTCGTCTCATA<br>GGTAACCAGGCGTCCGATAC

Table S1. Primers used for RCS rat qPCR analyses.
